# Lanthanides Release and Partitioning in Municipal Wastewater Effluents

**DOI:** 10.3390/toxics10050254

**Published:** 2022-05-17

**Authors:** Patrice Turcotte, Shirley Anne Smyth, François Gagné, Christian Gagnon

**Affiliations:** 1 Water Science & Technology, Environment & Climate Change Canada, 105 Rue McGill, Montréal, QC H2Y 2E7, Canada; patrice.tucotte@ec.gc.ca (P.T.); francois.gagne@ec.gc.ca (F.G.); 2 Science & Risk Assessment, Environment & Climate Change Canada, 867 Lakeshore Rd, Burlington, ON L7S 1A1, Canada; shirleyanne.smyth@ec.gc.ca

**Keywords:** rare earth elements, wastewater, partitioning

## Abstract

The use of lanthanides is increasing in our society, whether in communication technologies, transportation, electronics or medical imaging. Some lanthanides enter urban wastewater and flow through municipal wastewater treatment plants (WWTPs). However, little is known about the effectiveness of treatment processes to remove these elements and the concentrations released in effluents to receiving waters. The main objective of this study was to investigate the fate of lanthanides in various wastewater treatment processes. A secondary objective was to better understand the fate of medical gadolinium (Gd) complexes; anthropogenic inputs were differentiated from geological sources using an approach based on concentration normalization with respect to chondrite Post-Archean Australian Shale (PAAS). The hypothesis was that most lanthanides, especially of geological origin, are associated with the particulate phase and could be efficiently removed by WWTPs. To monitor these elements in different WWTPs, various urban influents and effluents from simple aerated lagoons to advanced treatments were sampled in Canada. The results showed that the rates of lanthanide removal by treatment processes decrease with their atomic number; from 95% for cerium (Ce) to 70% for lutetium (Lu), except for Gd, which was minimally removed. The normalization approach permitted the determination of the origin of Gd in these wastewaters, i.e., medical application versus the geological background. By distinguishing the geogenic Gd fraction from the anthropogenic one, the removal efficiency was evaluated according to the origin of the Gd; nearly 90% for geogenic Gd and a rate varying from 15% to 50% in the case of anthropogenic Gd. The processes using alum as the flocculating agent had the highest removal efficiency from wastewater.

## 1. Introduction

Lanthanides form a homogeneous family of rare earth elements (REE), from lanthanum (La) to lutetium (Lu) and typically with an oxidation state of III. Of similar geochemical properties in the aquatic environment, they are rather insoluble, react with phosphates and carbonates and form complexes with iron oxides and organic matter [1,2]. A peculiarity of the lanthanides is the contraction of the atomic radius following the increase in their atomic number (z) [3]. Cerium (Ce) and europium (Eu) have additional oxidation states: Eu (II) and Ce (IV), making them sensitive to redox potential changes [4]. Lanthanides are unique elements that have physical properties that make them necessary in various technologies, such as communication technologies, computer science, transport electrification, electronics, petroleum product refining and medical imaging [5,6]. Lanthanides may be released into the environment, particularly after being used for medical imaging, paints, oil/gas additives, and electronics, for example, and can be found in municipal wastewaters as with other lanthanides that have a geological origin. Gd is a special case of urban REE: it is largely used in medical imaging as a contrasting agent, which is excreted by the patient and enters urban wastewaters [7,8,9,10]. Gd is administered in the form of stable organic complexes to the patient prior to the nuclear resonance imaging scan and then excreted as complex forms, which ends up in domestic wastewaters. These stable Gd complexes are very polar and water soluble in contrast to geological Gd and have a relatively long half-life [9]. Bau et al. [7] demonstrated their presence by the use of a shale-normalized concentration of lanthanides approach and Gd anomaly calculations, as described below. In addition to these medical Gd, other lanthanides from anthropogenic sources were also found in contaminated aquatic environments. Among studies reporting the occurrence of lanthanides from anthropogenic sources, Kulaksis et al. [8] reported enrichment in anthropogenic La and samarium (Sm) in the Rhine River, where these lanthanides were used as catalysts in petrochemical industries [8].

In an attempt to differentiate between natural/geological occurrences of REEs, a normalization approach was devised [11,12]. The relative proportion of lanthanides to each other in chondrites, the parent rocks where most REEs are found, are relatively constant and are used to determine the origin of REEs found in miscellaneous samples [12]. Thus, the normalization of lanthanides concentrations is based on the ratio of measured concentrations of the test sample to those of the chondrite. The distribution of these normalized concentrations plotted versus atomic number follows a smooth distribution. The chondrite Post-Archean Australian Shale (PAAS) was chosen for this study [10,11,13]. For an unaltered natural sample, normalized lanthanides proportions are sufficiently stable that it is possible to estimate a normalized concentration (geogenic concentration) of one of these from its neighbors. For a given lanthanide, a ratio of the normalized measured concentration to the estimated concentration different than 1, is theoretically considered as a geochemical anomaly and an indication of the anthropogenic contamination. For the Gd, Rabiet et al. [14] considered that to have an anomaly, an experimental ratio greater than 1.4 was needed. This anomaly made it possible to highlight the presence of anthropogenic Gd associated with medical imaging activities [7,9,10,15]. Eu and Ce anomalies can also be associated with variations in redox conditions [2,4] since they possess more than one oxidation state (Eu^2+^, Ce^4+^). The lanthanides’ concentration and their relative proportion make it possible to understand a series of natural geochemical processes, such as erosion and even distinguish those natural processes from anthropogenic sources [2,12,16,17].

Contaminants released into municipal wastewater are removed at treatment plants with various efficacy based on treatment types and technologies. Although municipal WWTPs were originally designed to remove suspended solids, and reduce biochemical oxygen demands and ammonia, a number of contaminants are also removed to some degree. Metals are well known to be efficiently removed at WWTPs during solids separation processes, i.e., associated with the particulate phase. However, little is known about the fate of lanthanides in various treatments. The hypothesis is that most lanthanides, especially of geological origin, are associated with the solid phase and could be efficiently removed at WWTPs, which facilitate the settling of particulate materials. Questions remain for anthropogenic medical Gd, which is more in soluble forms and could thus escape treatment processes.

One of the objectives of this study was to evaluate the lanthanide concentrations and proportions of the dissolved phase (<0.45 um) in urban releases and to compare these releases to concentrations found in natural waters. In addition, this study aims to evaluate the lanthanide removal efficiency rates of six different treatment plants that use different processes. The issue of gadolinium being used in medical imaging is looked at according to its solubility and its stability, and the nature of the processes of the WWTPs.

## 2. Materials and Methods

### 2.1. Wastewater Sampling Collection

Samples of wastewater raw influent and final effluent were collected from 6 WWTPs of different treatment technologies across Canada from May to August 2018, duringdry weather flow conditions. Various types of treatment were investigated and included aerated lagoons (AL), secondary biological treatments using conventional activated sludge (ST), and advanced biological nutrient removal treatments with tertiary filtration (AT) (Table 1). Both influent and effluent samples were collected for three consecutive days (*n* = 3) using Hach Sigma 900 refrigerated autosamplers (Hach Company, Loveland CO, USA) to obtain 24-h equal volume composite samples of 200 mL every 15 min and to better consider effluent fluctuations. Influent and effluent samples were collected concurrently (i.e., no consideration of hydraulic retention time). Wastewater samples were subsampled in pre-cleaned 1 L high-density polyethylene bottles and shipped on ice to the laboratory. Subsamples of influent and effluent were transferred to 50 mL polypropylene tubes (Sigma-Aldrich TPP Centrifuge tube). Dissolved concentrations were determined following filtration on 0.45 µm membranes (Millipore Sigma Stericup (S2HVU0RE) Fisher Scientific) and transferred to 50 mL tubes. All subsamples were preserved with concentrated HNO_3_ (Baseline grade, SeaStar, Vancouver, Canada) for a final concentration of 2%. Subsequently, H_2_O_2_ 30% (SCP) was added to the subsamples for a final concentration of 2%. Prior to ICP analysis, the samples were heated at 70 °C for 24 h.

In addition, surface water samples were collected from the St. Lawrence River and Athabaska River, Canada, which were selected for their contrasting low (1.6 mg/L) and relatively high (11–26 mg/L) suspended particle matter (SPM), respectively (Table 2). 

### 2.2. Analysis in Wastewaters

Lanthanides analyses of urban wastewater samples were performed by argon ion plasma-mass spectrometry (ICP-RQ Thermo Scientific) at high sensitivity (LOD ≤ 0.05 ng/L). The plasma power was fixed at 1550 watts. The optimization was performed according to the manufacturer’s specifications: CeO/Ce < 2%, Ba^2+^/Ba < 3% and sensitivity for U 238 > 330 kcps/ppb. Calibration was achieved using standard ion solutions from SCP Science (Baie-d’Urfée, Montréal, Canada). By serial dilutions, solutions of 0.1, 0.5 and 1.0 μg/L were prepared in 1% HNO_3_ (Baseline grade, SeaStar, Vancouver, Canada). The lanthanides isotopes used for ICP-MS analysis and the determination of oxide and hydroxide interferences formation were performed as described by Merten and Büchel [18]. The analytical reproducibility, expressed as coefficient of variation, and the analytical exactitude, and recovery rate relative to a known concentration of reference material, were evaluated with the natural river water standard SLRS-6 (CNRC-NRC, Canada), for which lanthanides concentrations were evaluated by an inter-laboratory analysis [19]. The reproducibility, expressed as coefficient of variation, was ≤5% for all lanthanides and the exactitude was between 92% and 103%. Suspended particle matter (SPM) was analyzed by Environment and Climate Change Canada’s National Laboratory for Environmental Testing according to standard methods [20].

### 2.3. Calculation of Gd Anomalies and Removal Rate

Anthropogenic Gd in urban wastewater was determined by the calculation of Gd anomalies, an approach that estimates the geogenic concentration of Gd in a non-impacted sample based on neighboring lanthanides distribution. These calculations were made from the concentrations of lanthanides normalized (N) with the PAAS chondrites [10,11]. In this study, the neighbor elements Nd and Dy were used to estimate the geogenic concentration of Gd [10] (Equation (1)).

Geogenic Gd_N_ = 0.4 × Nd_N_ + 0.6 × Dy_N_ (1)

Normalized anthropogenic Gd concentrations in urban influents and effluents were estimated from the calculated geogenic Gd concentrations and those measured (Equation (2)). In an urban discharge, positive (>1 or even 1.4 as recommended by Rabiet et al. [14]) Gd anomalies (Equation (3)) are associated with the use of organic Gd complexes in medical imaging [7,9,10,14,15]. With this calculated (Equation (1)) geogenic normalized concentration, we estimated the concentration of anthropogenic Gd (Equation (2)) and the Gd_N_ anomaly in influent and effluent samples (Equation (3)) and the efficiency rate of treatment stations for anthropogenic Gd.

Gd_N_ anthropogenic = measured Gd_N_ − Geogenic Gd_N_ (2)

Gd_N_ anomaly = measured Gd_N_/Geogenic Gd_N_ (3)

## 3. Results & Discussion

### 3.1. Total and Dissolved Lanthanide Concentrations

Table 3 shows concentrations of total and dissolved (<0.45 µm) lanthanides measured in urban influents and effluents from six different WWTPs. The sum of the total REEs in influents varied from approximately 900 to nearly 7000 ng/L and between 160 and 330 ng/L in effluents. These total concentrations were in the same range as those measured in surface water collected from the St. Lawrence River upstream, near the city of Montreal (QC, Canada), which was of the order of 400 ng/L (Table 2). In contrast, higher concentrations were observed in the Athabaska River (AB, Canada), where the summations of total lanthanides ranged from 950 to 2400 ng/L and were associated with an increase in suspended particle matter (Table 2). Klaver et al. [21] noted similar concentrations (∑REE 678 ng/L) in surface water from the Rhine-Meuse River system in the Netherlands.

Total lanthanides concentrations released in effluents did not show any relationship with the process types used in municipal wastewater treatment plants (Table 1). Concentrations of dissolved lanthanides (<0.45 µm) in effluents varied between 102 and 189 ng/L (Table 3). These latter concentrations included dissolved Gd, which comes mainly from Gd complexes used in medical imaging [15] and which can represent nearly 80% of the dissolved REE concentration. For all wastewater samples, in both total and dissolved fractions, the Gd anomalies were greater than 1.4, the threshold set by Rabiet et al. [14]. When dissolved Gd is excluded, the concentrations of REEs in the dissolved phase among the WWTPs varied between 18 and 65 ng/L only.

**Figure 1:**
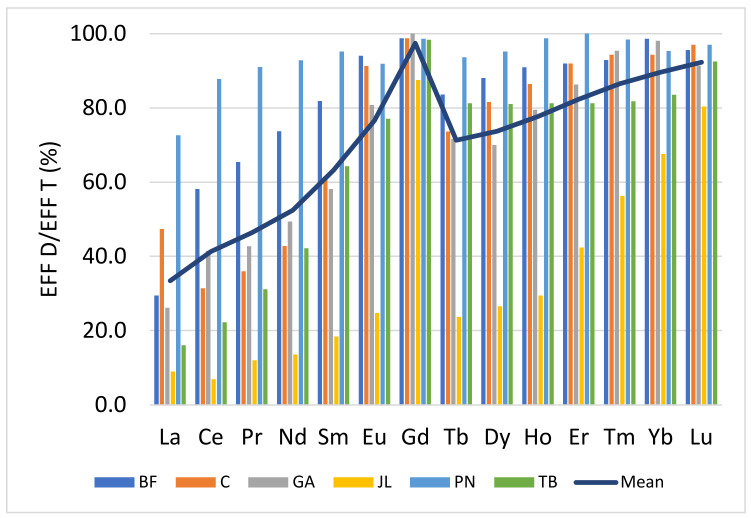
Dissolved proportion of lanthanides in effluents (*n* = 3) from various municipalities in Canada.

**Figure 2:**
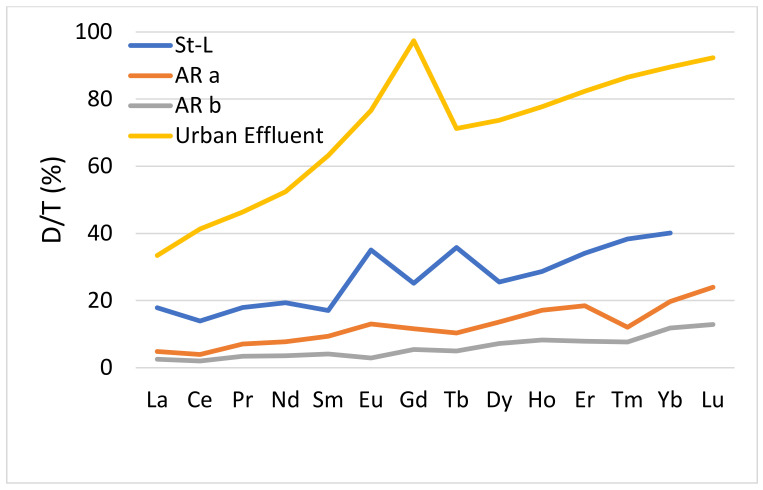
Dissolved proportion of lanthanides in St. Lawrence River (St-L), Athabasca River (AR) and the mean effluents from various municipalities in Canada.

Figure 1 shows the proportion of the dissolved phase, for each lanthanide, in effluents. Although the ratios between phases are different from one plant to another, the same trend is observed with an increase of it with the atomic number for all plants., except for the case of Gd. For La and Ce, the average proportion of the dissolved phase was 40%, while for certain heaviest lanthanides, Ho, Er, Tm, Yb and Lu were >80%. The comparative proportions of lanthanides in the dissolved phase with natural rivers were determined (Figure 2). The proportions of dissolved lanthanides in natural waters were much lower (<15%) than those found in urban discharges for all lanthanides (Table 1). Hence, the anthropogenic sources of lanthanides increase the proportion of dissolved elements in urban effluent compared to the proportion observed in rivers. These results imply that the contribution of urban discharges in the effluent receiving waters should show enrichment in heavy REE (HREE: Eu to Lu) and an increase in the proportion of the dissolved for HREE. Kulaksiz et al. [9] and Hissler et al. [10] observed this enrichment in HREE in surface waters near an urban discharge, which they characterized by a decrease in the ratio Nd_N_PAAS/Yb_N_PAAS. The results also indicated a decrease in this ratio between elements in effluents and those from natural waters (Table 2 and Table 3). These ratios in whole effluents varied from 0.12 to 0.64, while they were estimated to be close to 1.2 in analyzed water samples from rivers. Those ratios with the dissolved fraction varied from 0.05 to 0.15 in effluents, while in natural waters, they varied rather from 0.4 to 0.6. Other studies monitoring concentrations of lanthanides in surface waters impacted by urban discharges have noted the same trend, i.e., deviations from the geological backgrounds [9,10,22,23].

**Figure 3:**
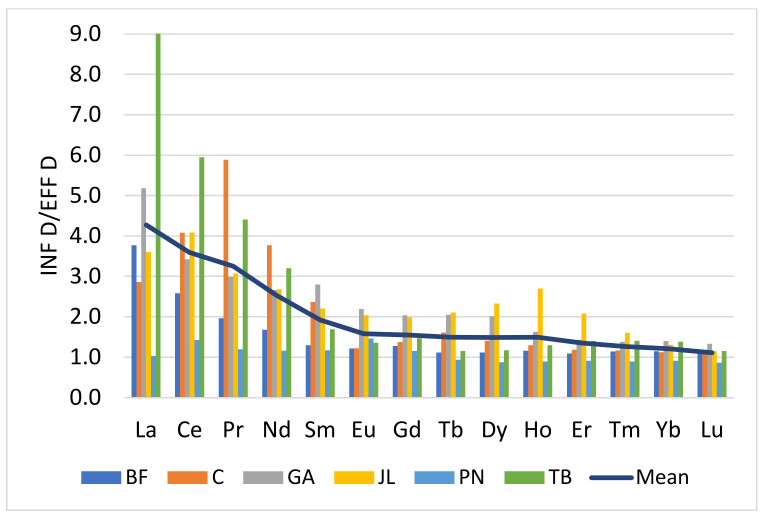
Ratio of the dissolved concentration in influent (INF D) and effluent (EFF D) for each lanthanides in various treatment plants.

HREEs tend to form more stable complexes with ligands available in the medium, namely Fe–Mn oxides, colloids and organic ligands [6]. These properties affect the distribution of REEs between the particulate and dissolved phases. The ratios of the dissolved concentrations between influents and effluents showed a decrease following the atomic number (Figure 3). For La and Ce, these ratios were close to 4 while those of Yb and Lu, were near to 1. A ratio greater than 1 suggests that part of the dissolved phase of the influents is complex and transferred to the particle phase. The ratio near a value of 1 indicates that the dissolved phase of the influent is little affected by the treatment process of the plant and that lanthanides tend to remain in the dissolved phase. These results also show that light-dissolved REEs (LREE: La to Sm) tend to be complexed with particles during wastewater treatment. Several authors have noted that the adsorption of LREEs occurs preferentially on particles while HREEs tend to remain in solution [3,6,17,24]. It is also known that the solubility of HREEs is increased by the speciation of lanthanide complexes with carbonates and bicarbonates, to the detriment of LREEs [25,26,27]. As a result, the distribution of particulate REEs is enriched in LREE while the HREE in the dissolved phase. In the case of urban effluents, this resulted in a normalized lanthanides distribution enriched in HREE. The results also showed that the treatment of urban wastewater is less effective in proportion to HREEs, and these are mainly found in the dissolved phase of the discharged effluents.

### 3.2. Removal Efficiency Rate at Wastewater Treatment Plants

**Figure 4:**
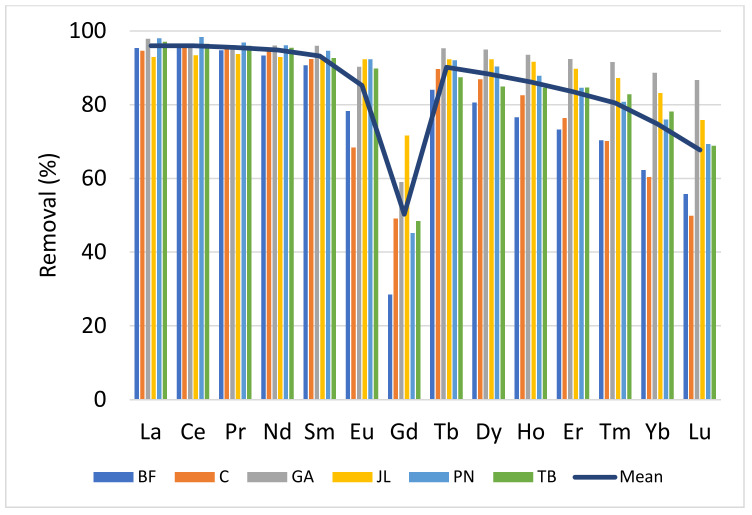
Removal efficiency of total lanthanides in various municipal wastewater treatment plants.

Based on the measured lanthanide concentrations in influents and effluents, the removal efficiency of the different WWTPs was estimated for each total lanthanide (Figure 4). A constant decrease in the removal efficiency was observed from the LREE Ce to HREE Lu. The exception of Gd in urban discharges was associated with medical imaging techniques that use soluble and stable organic complexes of Gd. The average removal rate was 95% and 70% for Ce and Lu, respectively, while it was only 50% for Gd. Pinter et al. [28] have recently obtained similar results on the removal rate (85%). The decrease in removal efficiency followed the increase in the atomic number of the lanthanides and, implicitly, the decrease in the atomic radius. Observed removal efficiency rates did not show any clear relationship with the treatment process types as all WWTPs removed more than 90% of the total suspended matter (Table 1 and Table 4). On the other hand, two wastewater treatment plants, GA and JL, using alum (Table 1) as a flocculating agent to reduce the concentration of phosphates (PO4^3−^), showed greater removal rates for dysprosium (Dy) and heavier lanthanides compared to the other WWTPs. The ionic radius of these lanthanides decreases with atomic number, are more electronegative and tend to form more stable complexes [4,6,12]. It is reasonable to consider that the alum entrains a part of the lanthanides complexed by organic matter in the particulate phase of an urban discharge where more than 98% was removed at all the WWTPs.

### 3.3. Anthropogenic Gd in Urban Influents and Effluents

Table 4 shows concentrations of total (including geogenic) and anthropogenic Gd associated with medical imaging, and the removal efficiency of WWTPs for both Gd origins. The efficiency of the processes for removing geogenic Gd ranged from 85% to 96%. These average levels were similar to neighboring lanthanides of Gd, namely Sm (93), Eu (85) and Tb (90) (Figure 4). For anthropogenic Gd, associated with medical imaging, removal rates varied between 23% and 54% (Table 4), and there were no trends with the process type (exception with alum) observed, suggesting that the release of anthropogenic Gd is impervious to the type of the wastewater treatment. Daily discharge of Gd was, however, very different from day 1 to day 3 with relative variation coefficients between 14% and 75%. By calculating uncertainties (relative standard deviation) on concentrations, these daily variabilities were taken into account for the calculation of the uncertainty associated with the removal rate for Gd complexes. Other studies have also reported that treatment processes retain only a small fraction of the organic complexes of Gd MRI [29,30]. Künnemeyer et al. [29] investigated these complexes by liquid chromatography coupled with an ICP-MS (LC hyphenated to ICP-MS sector field (SF)) along the different stages of an urban WWTP. They noted that, between each step of the treatment, there was a decrease in the amount of organic Gd complexes. One hypothesis, put forward by several authors to explain the removal of a part of this Gd complex, is that there would be a substitution of Gd in organic complexes by another metal (transmetalation), among others by Fe^3+^, Cu^2+^ and Zn^2+^, which are present in urban influents [29]. Rabiet et al. [31] showed that the addition of Fe^3+^, Cu^2+^ or Zn^2+^ significantly displaced the Gd of a complex used in medical imaging, Gd-DTPA, in an aqueous solution. The Gd^3+^ thus released would be segregated by the urban discharge matrix, but its fate in treatment processes remains little known [31]. These last three metals are present in urban wastewater and Fe^3+^ is often added as a flocculating agent to remove phosphates [32]. To support this hypothesis, Puttagunta et al. [33] calculated the stabilities of different organic complexes with different metals and those with Fe, Cu or Zn in water were reported as stable. However, this study was not carried out in an urban discharge or in an environmental context. Despite the low potential impact on the overall fate of Gd in wastewaters, it is reasonable to consider this hypothesis for further investigation of the fate of Gd complexes. Finally, we observed that the two treatment processes, GA and JL, which use alum as a flocculent to remove phosphorus and organic matter, had the highest removal efficiency at just over 50%. We can hypothesize that alum can segregate some of the organic Gd complexes used in medical imaging, but this hypothesis should be validated in a laboratory.

## 4. Conclusions

While total concentrations measured in treated wastewater effluents were comparable to those measured in surface water, the dissolved fraction was higher in effluents. Treatment plants were efficient (75–90%) in removing the particulate fraction of lanthanides, being naturally occurring as particles. LREEs were observed to be more efficiently removed by treatment plants when compared to HREEs. The element Gd was an exception, being significantly released as soluble complexes. The study showed that the process efficiency rate for removing geogenic Gd was on average 91%, which was similar to the estimated rates for neighboring lanthanides. The Gd used in medical imaging, however, was removed to the lowest extent. As an explanatory fact, only a small portion of it was associated with the particulate phase of influent wastewater. The data did not show any relationship between their removal efficiency and the type of treatment process. However, processes using alum as a flocculent to remove phosphorus showed a tendency for higher efficiency in HREE removal. The same trends were noted, being a higher removal of medical Gd in treatment plants using alum. To confirm this trend and better explain the fate of lanthanides in treatment plants, further studies will be needed.

This study showed that the proportion of dissolved lanthanides (<0.45 µm) in urban effluents is much greater than that found in natural waters and that there is an enrichment of HREE. Future work to determine the toxicity of lanthanides should consider these results, especially the phase partitioning of lanthanides released from effluents. There is a considerable lack of ecotoxicity data from environmentally relevant exposure for the evaluation of the potential hazard of lanthanides in aquatic ecosystems [34]. Finally, it would be interesting to ask the question of if the measured dissolved lanthanides are in colloidal forms, and if they are colloidal forms, what is the nature of their ligands?

## Figures and Tables

**Table 1:** Wastewater treatment process types.

					Inputs (%)	
Plant ID	Wastewater Treatment Plant Type	Hydraulic Retention Time (days)	Coagulant for Phosphorus Removal	Average Daily Flow (m^3^/day)	Residential	Industrial/ Commercial/ Institutional
BF	Secondary actived sludge	1.3	Ferrous	34,062	45	55
C	Secondary actived sludge	N.A.	None	16,928	90	10
GA	Secondary actived sludge with sand filtration	N.A.	Alum	27,125	65	35
JL	Aerated Lagoon	15–18	Alum	15,229	80	20
PN	Advanced biological nutrient removal	1.3	None	11,723	90	10
TB	Aerated Lagoon	20	Ferric sulfate	18,242	90	10

All samples collected May–August 2018. N.A.: not available.

**Table 2:** Concentrations of lanthanides and suspended particle matter (SPM) in waters of natural rivers, St. Lawrence River (St-L) and Athabasca River (AR), total (T) and dissolved (D) and the ratio Nd_N_/Yd_N_.

	REE (ng/L)																
River	La	Ce	Pr	Nd	Sm	Eu	Gd	Tb	Dy	Ho	Er	Tm	Yb	Lu	ΣREE	Nd_N_/Yd_N_	SPM (mg/L)
St-L T	91.7	177	22.2	82.6	15.4	3.33	12.8	2.05	10.9	1.92	5.54	0.81	5.32	N.A.	431	1.29	1.6
St-L D	16.4	24.6	3.98	16.0	2.62	1.17	3.22	0.74	2.78	0.55	1.89	0.31	2.13	N.A.	76.3	0.62	
AR.a T	174	360	48.0	189	43.7	9.46	40.9	5.90	34.8	6.50	17.8	2.39	13.7	2.07	946	1.15	11
AR.a D	8.44	14.2	3.38	14.5	4.09	1.23	4.76	0.61	4.72	1.11	3.28	0.29	2.70	0.50	63.3	0.45	
AR.b T	444	937	120	482	108	22.6	97.1	14.3	75.1	14.7	39.2	5.09	29.6	4.62	2388	1.35	26
AR.b D	11.1	18.8	4.08	17.2	4.39	0.64	5.24	0.70	5.42	1.21	3.08	0.39	3.50	0.60	75.8	0.41	

St-L: Saint Lawrence River. AR: Athabasca River.

**Table 3:** REE concentrations in total (T) and dissolved phase (D) of wastewater influents (INF) and effluents (EFF) (*n* = 3) from six treatment plants in Canada. Gadolinium anomaly calculations in wastewater and ratio Nd_N_/Yd_N_.

Plant		ng/L/(STDS)																Gd
ID	Fraction	La	Ce	Pr	Nd	Sm	Eu	Tb	Gd	Dy	Ho	Er	Tm	Yb	Lu	∑ REE	Nd_N_/Yd_N_	Anomaly
BF	INF T	226	248	25.9	109	24.1	6.22	3.78	222	20.0	3.89	11.2	1.58	12.6	1.95	916	0.72	12.3
	(STDS)	(74)	(15)	(2.6)	(17)	(4.3)	(0.55)	(0.63)	(49)	(1.6)	(0.16)	(0.4)	(0.03)	(0.36)	(0.05)			
	INF D	10.5	10.9	1.36	7.27	2.24	1.35	0.60	158	3.88	0.91	3.00	0.47	4.75	0.86	207	0.13	58.2
	(STDS)	(0.83)	(2.1)	(0.19)	(1.01)	(0.45)	(0.17)	(0.16)	(73)	(0.66)	(0.13)	(0.33)	(0.04)	(0.34)	(0.05)			
	EFF T	11.7	16.3	1.74	8.98	2.38	1.54	0.56	200	3.80	0.96	3.02	0.49	5.37	0.91	257	0.14	72.1
	(STDS)	(1.3)	(0.4)	(0.05)	(0.34)	(0.24)	(0.08)	(0.03)	(48)	(0.29)	(0.05)	(0.19)	(0.01)	(0.36)	(0.01)			
	EFF D	3.11	6.33	0.89	5.36	1.84	1.27	0.50	156	3.42	0.83	2.76	0.43	4.69	0.82	189	0.10	66.9
	(STDS)	(0.31)	(0.98)	(0.14)	(0.94)	(0.35)	(0.09)	(0.10)	(73)	(0.59)	(0.12)	(0.27)	(0.03)	(0.23)	(0.04)			
C	INF T	302	565	77.3	333	77.3	22.9	11.3	230	58.9	11.4	31.8	4.29	28.4	5.02	1758	0.97	4.3
	(STDS)	(133)	(267)	(34.7)	(143)	(29.5)	(3.6)	(2.7)	(37)	(17.9)	(3.2)	(7.9)	(0.95)	(5.2)	(0.71)			
	INF D	16.2	23.4	3.40	16.1	5.88	7.24	1.17	117	7.72	1.99	7.51	1.28	11.3	2.51	223	0.12	21.3
	(STDS)	(3.0)	(5.3)	(0.81)	(3.8)	(1.07)	(0.35)	(0.12)	(36)	(0.66)	(0.10)	(0.31)	(0.04)	(0.3)	(0.05)			
	EFF T	22.0	30.0	7.21	26.0	8.56	8.05	1.38	159	8.85	2.23	8.17	1.40	11.9	2.63	297	0.18	23.7
	(STDS)	(5.5)	(7.2)	(4.33)	(4.2)	(1.24)	(0.47)	(0.21)	(58)	(1.31)	(0.24)	(0.61)	(0.08)	(0.7)	(0.15)			
	EFF D	7.69	7.35	1.23	6.89	3.62	6.61	0.86	115	6.29	1.72	6.90	1.21	10.6	2.44	179	0.05	27.9
	(STDS)	(0.19)	(0.45)	(0.08)	(0.30)	(0.17)	(0.04)	(0.07)	(41)	(0.47)	(0.08)	(0.35)	(0.06)	(0.6)	(0.11)			
GA	INF T	600	363	36.2	144	30.3	8.77	5.08	198	28.3	5.57	16.6	2.35	18.2	2.25	1459	0.66	8.0
	(STDS)	(142)	(118)	(14.1)	(57)	(12.1)	(2.21)	(1.78)	(148)	(11.3)	(2.18)	(6.3)	(0.92)	(8.4)	(0.87)			
	INF D	12.7	12.6	1.37	5.63	1.22	0.85	0.24	81.2	1.42	0.36	1.27	0.20	2.07	0.30	121	0.23	70.0
	(STDS)	(0.9)	(1.1)	(0.07)	(0.40)	(0.11)	(0.03)	(0.05)	(56.7)	(0.11)	(0.02)	(0.11)	(0.01)	(0.32)	(0.01)			
	EFF T	17.2	17.9	1.75	7.40	1.98	1.51	0.35	170	2.00	0.47	1.56	0.26	2.85	0.36	225	0.22	105.8
	(STDS)	(6.2)	(5.4)	(0.41)	(1.53)	(0.36)	(0.09)	(0.05)	(134)	(0.36)	(0.06)	(0.08)	(0.02)	(0.74)	(0.04)			
	EFF D	3.32	5.23	0.59	2.78	0.71	0.69	0.17	83.3	1.00	0.29	1.09	0.19	2.03	0.27	102	0.11	111.4
	(STDS)	(0.23)	(0.40)	(0.03)	(0.33)	(0.08)	(0.08)	(0.03)	(60.4)	(0.08)	(0.03)	(0.05)	(0.01)	(0.36)	(0.01)			
JL	INF T	1729	2723	316	1193	208	38.4	22.4	309	125	24.3	69.8	9.73	65.2	10.7	6843	1.52	2.2
	(STDS)	(329)	(419)	(50)	(205)	(34)	(5.5)	(3.1)	(141)	(18)	(3.6)	(10.4)	1.45	(8.6)	(1.4)			
	INF D	122	180	19.7	84.6	15.1	2.95	1.72	87.7	9.60	2.03	7.13	1.24	11.0	2.58	548	0.64	8.4
	(STDS)	(10)	(16)	(5.7)	(6.8)	(1.2)	(0.16)	(0.13)	(0.8)	(0.63)	(0.16)	(0.43)	(0.06)	(0.5)	(0.04)			
	EFF T	39.6	50.5	7.24	30.8	6.13	1.48	0.86	153	5.94	1.61	6.28	1.12	9.67	2.37	316	0.26	29.1
	(STDS)	(5.8)	(5.5)	(0.34)	(1.5)	(0.22)	(0.04)	(0.03)	(143)	(0.27)	(0.07)	(0.12)	(0.01)	(0.05)	(0.04)			
	EFF D	11.0	12.4	2.36	11.5	2.78	0.73	0.41	76.8	2.55	0.60	3.02	0.70	7.41	2.07	134	0.13	35.7
	(STDS)	(1.0)	(1.1)	(0.15)	(0.4)	(0.12)	(0.03)	(0.01)	(1.8)	(0.10)	(0.01)	(0.11)	(0.01)	(0.06)	(0.05)			
PN	INF T	993	920	86.1	332	61.3	14.0	7.93	142	44.8	8.76	26.5	3.94	28.7	4.58	2674	0.96	3.1
	(STDS)	(297)	(326)	(11.0)	(53)	(8.4)	(1.8)	(0.76)	(72)	(6.7)	(1.10)	(3.1)	(0.46)	(2.5)	(0.58)			
	INF D	19.7	15.2	2.71	12.9	3.30	1.07	0.63	77.7	4.33	1.06	4.09	0.76	6.89	1.41	152	0.16	23.6
	(STDS)	(2.2)	(1.6)	(0.23)	(0.5)	(0.07)	(0.01)	(0.03)	(23.5)	(0.11)	(0.03)	(0.22)	(0.03)	(0.10)	(0.02)			
	EFF T	14.8	19.0	2.94	13.9	3.67	1.44	0.55	88.3	3.59	0.94	3.73	0.66	5.96	1.18	161	0.19	30.3
	(STDS)	(2.2)	(2.4)	(0.33)	(1.7)	(0.95)	(0.89)	(0.01)	(64.7)	(0.19)	(0.04)	(0.11)	(0.02)	(0.13)	(0.01)			
	EFF D	14.3	13.3	2.46	12.0	3.14	0.98	0.59	76.6	4.12	1.05	4.10	0.74	6.57	1.36	141	0.15	24.5
	(STDS)	(1.8)	(1.6)	(0.25)	(0.6)	(0.15)	(0.04)	(0.03)	(24.7)	(0.19)	(0.05)	(0.15)	(0.03)	(0.39)	(0.03)			
TB	INF T	801	926	102	404	76.8	16.1	11.5	251	58.2	11.4	33.3	4.48	31.7	5.14	2732	1.06	4.4
	(STDS)	(183)	(320)	(38)	(145)	(26.6)	(5.36)	(4.42)	(93)	(18.6)	(3.48)	(10.3)	(1.25)	(7.41)	(1.12)			
	INF D	23.6	32.4	4.13	18.3	5.66	1.64	1.45	130	8.79	1.75	5.10	0.77	6.92	1.60	242	0.22	20.7
	(STDS)	(2.22)	(2.56)	(0.43)	(1.4)	(0.31)	(0.06)	(0.04)	(1)	(0.27)	(0.03)	(0.16)	(0.01)	(0.15)	(0.04)			
	EFF T	34.9	42.8	5.67	24.7	6.16	1.72	1.36	187	8.33	1.84	5.81	0.88	7.96	1.70	330	0.26	29.5
	(STDS)	(2.3)	(4.4)	(0.53)	(2.05)	(0.48)	(0.04)	(0.15)	(101)	(0.32)	(0.03)	(0.07)	(0.04)	(0.08)	(0.08)			
	EFF D	3.78	7.20	1.29	7.71	3.64	1.27	1.18	127	7.12	1.42	4.14	0.63	5.78	1.48	174	0.11	27.2
	(STDS)	(0.08)	(0.11)	(0.03)	(0.22)	(0.02)	(0.04)	(0.01)	(1)	(0.13)	(0.01)	(0.09)	(0.01)	(0.04)	(0.02)			

**Table 4:** Concentration of the geogenic Gd and Gd complex associated with medical resonance imaging (MRI) in influents and effluents. Efficiency rate for removing suspended particulate matter (SPM), geogenic Gd and that resulting from medical activities.

	Influent (ng Gd/L)		Effluent (ng Gd/L)		Removal (%)		
Plant ID	Geogenic	MRI	Geogenic	MRI	SPM	Geogenic	MRI (STDS)
BF	18	204	3	156	99	85	24 (10)
C	54	176	6	111	99	90	37 (14)
GA	25	173	1	80	99	96	54 (40)
JL	140	169	10	77	92	93	54 (18)
PN	45	96	3	75	99	93	23 (11)
TB	57	194	6	123	99	89	37 (5)

## Data Availability

Data supporting the results reported in the article can be found in the figures and tables included in this paper.
